# Role of Large Clinical Datasets From Physiologic Monitors in Improving the Safety of Clinical Alarm Systems and Methodological Considerations: A Case From Philips Monitors

**DOI:** 10.2196/humanfactors.6427

**Published:** 2016-09-30

**Authors:** Azizeh Khaled Sowan, Charles Calhoun Reed, Nancy Staggers

**Affiliations:** ^1^ School of Nursing Department of Health Restoration & Care Systems Management University of Texas Health Science Center at San Antonio San Antonio, TX United States; ^2^ Nursing Care Excellence Center University Health System San Antonio, TX United States; ^3^ School of Nursing and Department of Biomedical Informatics University of Utah Salt Lake City, UT United States

**Keywords:** large clinical data, audit log, physiologic monitors, clinical alarms, alarm fatigue, intensive care unit, nursing

## Abstract

**Background:**

Large datasets of the audit log of modern physiologic monitoring devices have rarely been used for predictive modeling, capturing unsafe practices, or guiding initiatives on alarm systems safety.

**Objective:**

This paper (1) describes a large clinical dataset using the audit log of the physiologic monitors, (2) discusses benefits and challenges of using the audit log in identifying the most important alarm signals and improving the safety of clinical alarm systems, and (3) provides suggestions for presenting alarm data and improving the audit log of the physiologic monitors.

**Methods:**

At a 20-bed transplant cardiac intensive care unit, alarm data recorded via the audit log of bedside monitors were retrieved from the server of the central station monitor.

**Results:**

Benefits of the audit log are many. They include easily retrievable data at no cost, complete alarm records, easy capture of inconsistent and unsafe practices, and easy identification of bedside monitors missed from a unit change of alarm settings adjustments. Challenges in analyzing the audit log are related to the time-consuming processes of data cleaning and analysis, and limited storage and retrieval capabilities of the monitors.

**Conclusions:**

The audit log is a function of current capabilities of the physiologic monitoring systems, monitor’s configuration, and alarm management practices by clinicians. Despite current challenges in data retrieval and analysis, large digitalized clinical datasets hold great promise in performance, safety, and quality improvement. Vendors, clinicians, researchers, and professional organizations should work closely to identify the most useful format and type of clinical data to expand medical devices’ log capacity.

## Introduction

Clinical alarm systems safety is a national concern in the United States [1-7]. The US Joint Commission issued a National Patient Safety Goal, NPSG.06.01.01, titled, “Improve the Safety of Clinical Alarm Systems,” which requires health care facilities to establish alarm systems safety as a hospital priority and to identify the most important alarm signals to manage [8].

Of all devices, physiologic monitors (also referred to as bedside or patient monitors) were associated with the highest number of alarms and deaths in the US Food and Drug Administration’s MAUDE (Manufacturer and User Facility Device Experience) database where a total of 566 alarm-related deaths were reported [9]. Past research identifies the high rate of alarms produced by physiologic monitors [6,10-17], and alarm-related issues continue despite device design improvements. This poses a particular challenge for meeting the Joint Commission safety goal. Current methods to track alarm issues and outcomes of practice changes are time-consuming and challenging. This paper offers an in-depth discussion of a more novel technique for analyzing alarm data, managing alarms, and evaluating results of alarm practice changes. This paper (1) describes a large clinical dataset using the audit log from the physiologic monitors, (2) discusses the benefits and challenges of using an audit log in identifying the most important alarm signals and improving the safety of clinical alarm systems, and (3) provides suggestions for presenting alarm data and improving the audit log in physiologic monitors.

Patient monitors are an essential component in critical care treatment processes. In recent years, improvements were incorporated into these monitors to facilitate the monitoring process, including (1) connection to smaller portable monitors; (2) larger monitoring displays; (3) multimeasurement modules to capture different variables such as cardiac output and mixed venous oxygen saturation; (4) histograms and tabular views for trended data; (5) wireless transmission of bedside monitor data to central station monitors, other bedside monitors, hospital servers, and communication devices such as phones and pagers; (6) integration of smart alarms such as delay in alarm announcement; (7) integration of clinical protocols, such as detection and treatment of sepsis; and (8) a variety of alarm tone sounds and displays of color-coded messages based on alarm priority.

Most important, modern physiologic monitors now have the capability to log triggered alarms with associated data. On the basis of available technical features, some can log hundreds of thousands of data points and send them to large clinical datasets. Unfortunately, these datasets are rarely used for predictive modeling, personalized treatment, capturing unsafe practices, or guiding quality initiatives, yet a growing recognition exists among health care organizations, federal agencies, and health care policies on the importance of large clinical datasets [18,19].

A significant body of research exists on clinical alarm safety. The majority of studies used structured observations or field notes to quantify the volume and types of alarms in intensive care units (ICUs) [10,15-17,20,21], cardiac telemetry units [22], adult medical surgical units [23], pediatric medical units [24], and emergency departments [4,25]. Although a commonly used approach, these techniques can be problematic. The validity of observations depends highly on observers' knowledge and skills including the knowledge of the phenomenon, the intensity (volume and priority level) of the alarms triggered by different devices, ability of the observer to manage that intensity, and the number of variables under observation. Variables can go well beyond simple quantification to include identifying all alarming devices with the associated numbers and types of alarms, clinician response to alarms, sequence of alarms, patient condition during the alarms, and the duration of the alarm or clinician response time. The shortcomings of the observation technique also include cases where too many alarms or simultaneous or overlapped alarms affect the precision of the observation. This is specifically true for observations taking place on day shifts when patient procedures and alarms are greater in volume. The use of non–health care professionals as observers, not uncommon in these types of studies, can also limit the type and scope of data being collected [24]. Additionally, some alarm events cannot be captured by human observations because alarms are displayed based on priority chains, as described in the following sections of this paper. Thus, a need exists for more objective, complete, and comprehensive data to quantify alarms generated by monitoring devices. The use of alarm audit data could fulfill that need.

A few studies on alarm systems safety were found to use the available data from physiologic monitors to measure the actual number of clinical alarms. Two of these studies retrieved and used the audit log [14,17]. Other studies transferred these data from the monitors to a different database using software [11,12]. Nevertheless, none of these studies (1) addressed techniques about using the large set of clinical data generated from monitoring devices to quantify alarms; (2) described elements of the logged data, benefits of using such data, and challenges faced on data storage, retrieval, and analysis; or (3) provided suggestions for improvement of the logged data in order to be a useful source for clinicians, researches, vendors, and policy makers. This paper addresses these gaps.

## Methods

### Use of an Audit Log in an Alarm Safety Project

In our previous projects on alarm systems safety we utilized data logged from physiologic monitors to quantify alarm rates in a 20-bed transplant cardiac ICU [15-17]. Our previous projects examined the effect of a change in physiologic monitors’ alarm parameters on decreasing the number of false and nonactionable alarms as well as improving nurses' perceptions and attitudes toward clinical alarms. The audit log data file was retrieved from the server of the central station monitor of the transplant cardiac ICU for alarm rates 10 weeks before and 10 weeks after the change in monitor parameters. The results of these projects, as previously published [15-17], showed a significant reduction in alarm rate. On the basis of our experience in data retrieval and analysis using the audit log of alarm data from our previous projects in the transplant cardiac ICU and from our current projects in other adult ICUs (surgical trauma, neuro, and medical ICUs), the focus of this paper is to discuss important methodological considerations in the use of audit data for future health and informatics projects.

### Description of the Setting and Physiologic Monitors

Our 4 adult ICUs have a total of 230 nurses and 155 beds and are equipped with Philips IntelliVue MX800 (Koninklijke Philips N.V, Amsterdam, the Netherlands) bedside monitors. The central station monitor is Philips IntelliVue Information Center iX, an information hub that allows patient information management at the bedside, unit, and hospital levels through information transferred from the networked bedside cardiac monitors. Both bedside and central monitors are capable of capturing, displaying, and storing real-time waveforms, parameters, and alarms and are wirelessly connected to the institution's electronic health record (Sunrise, Allscripts® Chicago, IL, USA).

Bedside monitors are hardwired to a switch, which automatically transfers the monitors’ data to the central station monitor (Information Center). The data are then routed to a Health Level Seven (HL7) interface that converts the device data into HL7 format and sends them to the electronic health record. Wi-Fi connectivity is only available when a transport or portable monitor or multimeasurement module (MMS X2) is used and is disconnected from the bedside monitor (host monitor).

The monitors generate (1) patient-related (physiologic) alarms and (2) inoperative or technical (INOP) alarms. Technical alarms indicate the monitor’s inability to appropriately measure physiologic parameters. Alarms are announced in 2 different areas in the bedside monitor, one for physiologic alarms and the other for technical alarms. When triggered, alarms flash on both the bedside and central station monitors. Monitors display the level of an alarm by (1) sound, (2) number of asterisks (*) for physiologic alarms or exclamation marks (!) for technical alarms, and (3) color of the message. Physiologic alarms have 3 levels of priority from advisory (*) to high (***), and technical alarms have 3 levels ranging from soft (with no exclamation mark assigned next to the alarm) to moderate (!!) to high (!!!). Severe physiologic alarms are displayed in red, whereas yellow reflects moderate-level physiologic alarms.

### Data Storage and Retrieval of the Audit Log of the Physiologic Monitors

The audit log is a chronological record of the alarms and clinicians’ interaction with the monitors. It is stored by and retrieved from the Information Center database. Storage period is only 90 days, and then the Information Center begins to overwrite the oldest data. Retrospective (oldest) data beyond 90 days will be lost or no longer accessible. The maximum data retrieval period at one time is 50 days and the minimum is 15 minutes. Therefore, at least 2 retrievals are necessary for 90 days’ retrospective logged data (ie, 50 days and 40 days).

There are 2 types of audit logs. The patient audit log is patient specific and can be searched using the patient’s first or last name, medical record number (MRN), or bed label. The unit audit log contains unit-specific data. Search categories are Alerts (alarms) and Actions (represent clinician navigation and interaction with the monitor). Alerts search criteria include Red Alarm, Yellow Alarm, Logged INOP, and Alert Sounds. Clinician actions include 21 types of actions or search criteria. Examples include Silence, Pause/Resume, Measurement On/Off, Alarm On/Off, Alarm Limit Change, Stand By On/Off, Admission/Discharge/Transfer, and Paced Status Changed. After selecting the desired type of audit log, unit, and search duration and criteria, the resulting file can be exported into Excel (Microsoft) format for analysis.

### Description of Audit Log Data

Clinicians and researchers select variables of interest to download from the audit log. Table 1 displays an example of selected cases of data extracted at the unit level (unit audit log). In our transplant cardiac ICU project, we categorized alarms as categorical and numerical. Categorical alarms do not have upper or lower limits and are displayed in the log data as generated or ended. Examples of categorical alarms include types of premature ventricular contractions (PVCs) such as multiform PVCs and Pair PVCs, Run PVCs High, AFIB (atrial fibrillation), and Irregular Heart Rate. All technical INOP alarms are also categorical, such as Check Patient ID, Check Equipment, Batt (battery) Empty, and Leads Off. Numerical alarms are signaled if the parameter value was above or below the current programmed limits. Examples of these alarms include RR (respiratory rate), HR (heart rate), Apnea, PVCs/min, ABP (arterial blood pressure), NBP (noninvasive blood pressure), PAP (pulmonary artery pressure), SpO_2_ (peripheral capillary oxygen saturation), and Desat (desaturation). Some alarms fall under both categories. For example, Apnea alarms can be displayed in three different messages: (1) “***Apnea generated,” indicates cessation of respiration for longer than the programmed apnea time, (2) “***Apnea X:YY” where X:YY represents the apnea duration in minutes and seconds, and (3) “***Apnea > 20 sec,” which means respiration has stopped for more than 20 seconds.

The alarm messages in the “Alarm and Action” column (Table 1) includes (1) the priority of the alarm based on the number of “*” or “!” signs next to the alarm, (2) name of the alarming parameter, (3) value of the parameter when the alarm was generated for parameters with numerical limits, (4) default or programmed settings of the numerical parameter, and (5) status of whether the alarm was generated or ended. Table 1 also presents examples of “Actions” that indicate clinician interaction with the monitor (eg, Silence, Resume All Alarms, Patient transferred, Patient category set to Adult).

**Table 1:** An example of a unit audit log.

Date	Bed label	MRN^a^	Alarm and Action	Device name^b^
4/20/14 0:00:00	9115-S1	0000000	**PAPd^c^ 18 >16 Ended.^d^	PIIC iX: ixsurv006
4/20/14 0:00:00	9115-S1	0000000	**PAPd 18 >16 Generated.^d^	PIIC iX: ixsurv006
4/20/14 0:00:00	9115-S1	0000000	Yellow alarm sound played.^e^	PIIC iX: ixsurv006
4/20/14 0:00:02	9115-S1	0000000	**ABPs^f^ 170 >160 Generated.^d^	PIIC iX: ixsurv006
4/20/14 0:01:05	9123-S1	0000000	***Desat^g^ 70 < 78 Generated.^h^	PIIC iX: ixsurv005
4/20/14 0:01:05	9123-S1	0000000	Red alarm sound played.^e^	PIIC iX: ixsurv005
4/20/14 0:01:12	9123-S1	0000000	***Desat 73 < 78 ended.^h^	PIIC iX: ixsurv005
4/20/14 0:01:16	9115-S1	0000000	**ABPs 168 >160 Ended.^d^	PIIC iX: ixsurv006
4/20/14 0:01:20	9123-S1	0000000	Silence.^i^	PIIC iX: ixsurv005
4/20/14 0:01:40	9115-S1	0000000	*Multiform PVCs^j^ Generated.^d^	PIIC iX: ixsurv006
4/20/14 0:01:40	9115-S1	0000000	Resume All Alarms.^i^	PIIC iX: ixsurv006
4/20/14 0:01:40	9117-S1	0000000	**RR^k^ 37 >30 Ended.^d^	PIIC iX: ixsurv006
4/20/14 0:01:40	9111-S1	0000000	Patient transferred to 9035-S1.^i^	PIIC iX: ixsurv006
4/20/14 0:01:42	9095-S1	0000000	Patient category set to Adult.^i^	PIIC iX: ixsurv006
4/20/14 0:01:43	9090-S1	0000000	Pacer algorithm set to Pacer Algorithm On.^i^	PIIC iX: ixsurv006
4/20/14 0:01:44	9123-S1	0000000	ECG^l^ Leads Off Generated.^m^	PIIC iX: ixsurv006
4/20/14 0:01:44	9123-S1	0000000	INOP^n^ sound played.^e^	PIIC iX: ixsurv006
4/20/14 0:01:59	9123-S1	0000000	ECG Leads Off Ended.^m^	PIIC iX: ixsurv006
4/20/14 0:02:00	9093-S1	0000000	Equipment Offline.^i^	PIIC iX: ixsurv006
4/20/14 0:02:00	9115-S1	0000000	**PAPd 18 >16 Generated.^d^	PIIC iX: ixsurv006
4/20/14 0:02:00	9093-S1	0000000	Equipment Online.^i^	PIIC iX: ixsurv006
4/20/14 0:02:00	9085-S1	0000000	ST: Al. Limits ST-V2^o^ High: 1.6 ST-V2 Low: −1.6.^p^	PIIC iX: ixsurv006
4/20/14 0:02:00	9117-S1	0000000	Arrhythmia Off.^q^	PIIC iX: ixsurv006
4/20/14 0:02:02	9115-S1	0000000	Pause All Alarms.^i^	PIIC iX: ixsurv006
4/20/14 0:02:02	9075-S1	0000000	Arrhy: Missed Beat Off.^r^	PIIC iX: ixsurv006
4/20/14 0:03:00	9085-S1	0000000	SpO_2_^s^: Desat Limit 78.^p^	PIIC iX: ixsurv006

^a^MRN is the medical record number and was presented as zeros for confidentiality purposes.

^b^Device name refers to the Information Center host name (eg, PIIC iX: ixsurv006).

^c^PAPd: pulmonary artery pressure diastolic.

^d^These are examples of yellow physiologic alarms.

^e^These messages appear if “Alert Sound” was selected as a search criterion from the Alerts category.

^f^ABPs: arterial blood pressure systolic.

^g^Desat: desaturation.

^h^An example of a red physiologic alarm.

^i^These are examples of clinicians’ actions. They depend on the “actions” selected from the search boxes.

^j^PVC: premature ventricular contraction.

^k^RR: respiratory rate.

^l^ECG: electrocardiographic.

^m^An example of INOP alarm.

^n^INOP: inoperative.

^o^ST-V2: a segment in the electrocardiogram.

^p^Examples of User Action–Alarm Limit Change.

^q^An example of User Action–Measurement Off.

^r^An example of User Action–Alarm Off; Arrhy: arrhythmia.

^s^SpO_2_: peripheral capillary oxygen saturation.

## Results

### Benefits of the Audit Log

Benefits of the audit log are many and are as follows:

#### Easily Retrievable Data at No Cost

The audit log dataset is easily retrievable at no cost. Persons with legitimate access to the data, such as researchers, clinicians, biomedical engineers, and device representatives, can perform the search and obtain the data within a few minutes without having to coordinate with the information technology department.

#### Tracking of Clinicians’ Interaction With the Monitor

Clinicians’ interaction with the monitor can be tracked using time stamps. Examples of clinicians’ actions include enabling or disabling alarms and/or measurements, silencing and pausing alarms, and changing alarms’ limits.

#### Complete Records of Data

All types of configured or programmed alarms are automatically recorded by the Information Center in the audit log and have no missing data. Furthermore, more complete records are available than with observational data, as the audit log can capture and display different values of the same parameter from different sources, such as ABPs (systolic) or ABPm (mean), if programmed by clinicians. These additional values provide an objective record of the number of alarms and likely better reflect sources of alarm fatigue. Duplicative alarms are easy identified. Overuse of alarms can be identified and targeted for elimination. Additionally, the Information Center can store different types of electrocardiographic (ECG) and non-ECG waves in graphic and tabular formats. This can be extremely valuable information for alarm annotation.

#### Evaluation of Quality Initiatives

Quality initiatives can be evaluated using audit data. The audit log can be used to evaluate the effectiveness of different interventions by comparing pre- and postintervention data [16,17]. For example, evaluations could occur after best practice education sessions on frequency and methods of changing electrodes and the difference in “leads off” alarms. Nurse adherence to different targeted interventions can be evaluated [16,17].

#### Identifications of Monitors Missing Required Parameter Changes

Managers can easily identify monitors missing any required parameter changes, as the audit log can identify specific bedside monitors missing the required adjustments. For example, in one of our previous projects [16], we found alarms on our audit log that we thought were disabled, such as Paired PVCs or Bigeminy PVCs. The audit log included the bed number of the monitor lacking the required changes.

#### Detection of Unsafe Limits and Inconsistent Practice

Any parameters changed to unsafe limits and inconsistencies in practice can be identified. Setting limits for each parameter across monitors can be easily tracked using audit data. Unit managers can then monitor whether alarm limits were adjusted safely for the patient's condition. Table 2 shows selected cases with variations in the lower limit setting for the Desat parameter ranging from 50% to 90%. Clearly, 50% is an unsafe lower limit for that parameter. This information can also be easily obtained from User Action–Alarm Limit Change search criterion.

Similarly, the audit log data may indicate inconsistencies in the priorities assigned to some parameters. For example, we found that a low priority was assigned to the HR (1 asterisk) and Batt Empty (1 exclamation mark) alarms in some cases, whereas these were a higher priority elsewhere (2 asterisks and 2 exclamation marks). This was despite the similarity in the value of the triggered alarm in the 2 priority cases in the HR limits (eg, “*HR 153>150 Generated” and “**HR 153 >150 Generated”).

#### Easier Comparisons Across Studies

Finally, comparisons across alarm studies may be easier, as alarms can be analyzed per patient days, bed, hours, or minutes, alarm parameter, and parameter priority. With the lack of published standards on reporting alarm rates, the audit log could allow easier comparison across studies on clinical alarm safety, specifically because different previous studies reported alarm rates using different units of analysis.

Benefits are obvious. In comparison with the observation technique, the use of the audit log allows cost-effective collection of alarm data, eliminates missing alarm data, safeguards the objectivity of the data, and, most important, allows unique discoveries from the collected information for analyses.

**Table 2:** An example of variations in setting the lower limit of the Desat (desaturation) parameter.

Date	Bed label	Alarm and Action	Device name^a^
7/16/14 4:57	9101-S1	*** Desat 89 < 90 Generated.^b^	PIIC iX: ixsurv007
7/28/14 21:44	9115-S1	*** Desat 87 < 88 Generated.	PIIC iX: ixsurv006
7/30/14 1:24	9109-S1	*** Desat 8 < 80 Generated.	PIIC iX: ixsurv006
8/11/14 11:59	9097-S1	*** Desat 44 < 50 Generated.	PIIC iX: ixsurv007
8/12/14 10:43	9113-S1	*** Desat 80 < 83 Generated.	PIIC iX: ixsurv006
9/5/14 21:38	9123-S1	*** Desat 0 < 78 Generated.	PIIC iX: ixsurv005

^a^Device name refers to the Information Center host name (eg, PIIC iX: ixsurv006).

^b^The three starts (***) indicate that Desat (desaturation) is a red or high priority alarm.

### Challenges in Analyzing the Audit Log Data

Challenges exist in analyzing the audit log retrieved from the Philips Information Center. One challenge is that data cleaning and analyses are time-consuming processes. For physiological (yellow and red alarms) and INOP technical alarms, the “alarms and action” cell (Table 1) includes 3 (for categorical alarms) to 6 (for numerical alarms) different variables about the alarm. These include alarm priority, name of the alarming parameter, value of the alarming parameter that initiated the alarm, the upper limit of the programmed setting, the lower limit of the programmed setting, and the status of the alarm (generated vs ended). Some technical alarms are displayed with no priority assigned to them (eg, ECG Leads Off). To export the Excel audit log file into IBM SPSS (IBM Corporation) for analysis, for example, numerical and categorical alarms need to be first separated into 2 files. Then, technical alarms without priorities need to be filtered out from the categorical data and entered after importing all other categorical data into SPSS. Likewise, numerical alarms with distinctive displays (without “>” or “<” signs, such as Apnea X:YY) also need to be filtered out from the numerical alarm Excel file and then entered after importing the data into SPSS. The latter 2 cases require rearranging the date column to provide trended, date-based alarm data.

Additionally, the generation and end times of an alarm event are logged as separate, unconnected events. Analyzing the duration of each alarm requires sorting the data per the MRN, bed, date, and device name, separating the generation from the end alarm times and then pasting correlated events together. Data sorting is necessary, specifically because the audit log records the end time of an alarm before the generation time for alarms that signaled for less than a second (see Table 1, rows 1 and 2). Tracking alarm duration is a critical factor indicating clinician response time to an alarm and also contributes to alarm fatigue, specifically for long alarms that keep beeping with no immediate attention. Clearly, attention to detail is required in data cleaning as missteps can result in data interpretation errors.

Finally, the available Information Center stores data only for 90 days and allows the retrieval of 50 days of data at a time. This limited storage and retrieval increases the required time for data downloads, data cleaning, and analyses if separate downloads are needed for retrospective studies. For example, we had to retrieve alarm data 3 times, 2 for 50 days and 1 for 40 days, in order to capture all data over the 20-week project period. According to the vendor, an option exists for a longer storage period with an additional purchase, but many sites may choose the more economical version.

## Discussion

### Considerations for Presenting Alarm Data

Previous research presented the number and types of alarms, limits of parameters, and changes in parameters’ limits [10-13,15-17,20-23,25-27]. This information is insufficient to inform contemporary quality initiatives on alarm safety. Alarms are announced based on monitor features and configuration as discussed below. The features below, which are usually absent from clinical alarm safety studies, must be explicitly discussed to understand alarm behaviors and for comparisons across studies.

#### Loss of Connectivity

Researchers and clinicians need to understand the data storage mechanism on servers from different vendors of cardiac monitors to estimate the number of alarms missed (if any) during any losses in connectivity. Data connection to the server can be lost in cases of hardware failure or system upgrade and maintenance. In our system, when connection to the server is lost, the data are saved in the bedside monitor and rerouted back to the server when the connection is restored. However, if a patient is disconnected from the bedside monitor and connected to the wireless transport monitor and the wireless device was out of range, data will be lost. Cases of connectivity loss are captured and recorded by our audit log. This allows the analysis and reporting of reliable data.

#### Indication of Latching Versus Nonlatching Alarms

When presenting alarm rates, duration, and corresponding alarm fatigue, researchers need to identify latching and nonlatching parameters. Some critical alarms are configured as “latching,” which are high-priority red alarms (***) that signal nonstop continuous audible sound even after the condition is no longer present, requiring a clinician to silence them (eg, asystole and ventricular fibrillation). For both latching and nonlatching alarms (where alarm indicators reset after the condition ends) when they are acknowledged and the condition is still present, the audible alarm will turn off as well as the alarm lamp but the flashing numeric will keep on as well as the audible reminder (if configured to do so). The audible reminder is recorded as a separate alarm event. Latching versus nonlatching alarms affect both the number of alarms and alarm duration.

#### Indication of Basic Versus Enhanced Alarms

It is equally important to identify whether alarms are set as basic (standard) versus enhanced. For example, in the arrhythmia analysis using our monitors, “Basic” capability allows the analysis and recording of 10 different arrhythmia alarms, for example, asystole, ventricular fibrillation, and ventricular tachycardia. The “Enhanced” arrhythmia analysis provides 13 additional alarms, for example, nonsustained ventricular tachycardia, supraventricular tachycardia, and run PVCs. Therefore, identifying the monitor configuration as basic or enhanced arrhythmia analysis would reflect the number of expected alarms.

#### Identification of Automatic Detection

Parameters set to automatic measurement or detection mode should be reported in alarm rates. This feature allows the monitor to detect measurements from different sources and decreases the number of false alarms. For example, automatic detection of respiration allows the monitor to adjust the detection of the respiration automatically, and the use of “Enhanced Asystole Detection” eliminates false asystole alarms.

#### Alarm Delays

Another factor to list is the use of “Smart Alarm Delay” and the mode of the delay, which delays an alarm based on the amount and duration over the set limit. This will eliminate the number of alarms for patients recovering from an alarm condition and appropriately decrease the total number of alarms.

Identification of automatic detection and alarm delay are very important to be reported given that some alarms may last for less than a second as shown in Table 1, which indicates lack of clinical significance.

#### Pausing and Silencing Alarms

Pausing and silencing alarms affect the duration of alarms. For example, some monitors allow “Pausing” alarms for 1 or 2 minutes or infinity (disabling the alarm). This also affects the number of false alarms. Another notable feature is that some systems allow “All Alarms Off for Yellow Alarms Only” and not for red alarms, whereas others allow this function for all types of alarms.

#### Priority Chain for Alarm Display

The priority chain of the alarm display affects the number of the announced alarms. The Information Center displays alarms based on 3 criteria: alarm sound, number of asterisks or exclamation marks in the message, and color of the message. Some situations inhibit the audible and visual indication of the alarm even when it is detected by the system and recorded in the audit log. These include cases of concurrent alarms where the system displays the most serious life-threatening event with highest priority based on a default priority algorithm using 3 chains (PVC alarms, beat detection, and rate alarm). All other alarms go to a display accessible by a drop-down list. Only the highest-priority alarm condition in each chain is announced. In cases of active high-priority alarm, the lower-priority alarms will not be announced. For example, when a Paired PVCs alarm is active and announced and another Pause alarm is detected, the Pause alarm will not be displayed because it is a lower priority. If another condition from another priority chain with equal severity is detected, the monitor will announce the more recent alarm. If the alarm is silenced by the nurse but the condition persists, the alarm message will still be displayed but without sound. The system first announces any unacknowledged red alarms, then any unacknowledged long yellow in the presence of any other yellow or INOP alarms, then short yellow alarms, then hard INOP technical alarms, followed by the soft INOP technical alarms (alarms with no priority assigned to them).

In cases of more than 1 alarm, an arrow to the right of the message on the central station monitor must be clicked to display a list of all active alarms with their times. A maximum of 10 alarms are displayed. In observation methods these alarms may be missed.

#### Alarms Not Amenable to the Changes (Hard Stops)

Monitors include default settings not amenable to changes by the clinician and only a monitor representative can change them. Examples of these settings are TachyClamp, BradyClamp, TachyExtract, and BradyExtract. Changing the default settings of all other alarms affects the alarm rate; therefore, researchers will want to indicate the types of alarms not amenable to clinician changes.

#### Audible Versus Inaudible Alarms

Studies need to identify and present the types of audible versus inaudible alarms [13]. For example, in our system there is no sound for soft INOP or technical alarms such as Noisy ECG. Although the audit log records the 2 types of alarms, audible alarms contribute more toward alarm fatigue.

#### Connecting Alarms to the Appropriate Settings and Reliable Monitoring Conditions

Different conditions may affect the number of alarms, specifically in cases of inappropriate settings made by the clinician or conditions affecting the reliability of the monitoring process. For example, clinicians must select the appropriate primary and secondary leads for the monitor to compute heart rate and to detect arrhythmias. The arrhythmia system automatically classifies patients’ beats. To decrease the chance of false alarms, nurses need to modify the ECG analysis and relabel any arrhythmia beats if they do not agree with the way the monitor is classifying beats. For patients with a pacer, nurses should make sure that the system is not counting pacer spikes as QRS complexes.

When ST and STE (ST-segment elevation) are both in use, redundant ST Elevation alarms will occur. Additionally, different values will be obtained because of the different measurement points (isotonic point and ST point are used for ST measurement and isotonic point and the J point are used for STE measurement). Thus, nurses need to adjust the ST measurement points for appropriate ST detection. Because STE alarms are patient specific, nurses need to set the 12 leads appropriately for each individual patient. Each ST lead has its own alarm limits.

In some conditions, monitoring some parameters is unreliable and may cause false nonactionable alarms. For example, ST monitoring is not recommended in cases when arrhythmias such as atrial flutter and fibrillation are present.

In the future and for the most accurate data, researchers will want to correlate alarm data to these conditions. None of the past studies on alarm safety correlated alarm data to whether appropriate monitor settings or reliable monitoring conditions existed.

### Suggestions for Improvement and Future Directions

The audit log is a combination of the current capabilities of the monitoring systems, the specific monitor configuration, and alarm management practices and clinician-monitor interaction. The recommendations below can improve monitoring systems practices and optimize audit log data for performance improvement and research.

First, improvement in capabilities of the monitors for data recording, storage, and presentation is recommended. Vendors need to enhance the standard recording, retrieval, and storage capabilities of monitors. Longer recording and storage periods are recommended. Adding the list of parameters (eg, HR, RR, PVC) to the Alerts search criteria would be very helpful for researchers and clinicians. Each alarm event (from generation to end) should be displayed as 1 event versus 2 events to more easily identify duration of the event. This would also help assessments on alarms lasting for more than a specific time period (eg, 1 minute). Additionally, the use of common nomenclature for alarm reporting between vendors is highly recommended to facilitate comparison across studies. This includes visual and audible alarm indicators, alarm behaviors, and meaning of parameters and alarms (eg, TachyClamp, basic vs enhanced).

Second, expand the tracking of user interaction with the monitor. Although monitors have some capabilities to track user actions, such as disabling or enabling alarms and measurement, they do not track screens visited. Tracking user interactions with the monitor via the visited screens could capture unsafe practices, common approaches in addressing alarms, best practices, work-arounds, and indicate clinician knowledge of the monitors’ capabilities. Previous studies used direct observation technique or surveillance cameras to capture clinician response to alarms [11,12], but few studies are available about how nursing practice and monitor configuration affect the number of alarms. For example, the use of the “Extending Alarm Pause Time Function” can extend the alarm pause time in cases of long procedures and decrease the number of false alarms.

Third, there is a need for expanding the audit log. Incorporating clinical data such as medications or laboratory values into the audit log could be extremely useful for more accurate alarm annotation. Monitoring devices and the audit log are currently based on univariate alarm algorithms where alarms are triggered based on the limits of one parameter. However, modern monitors allow detection of trended data (changes in a parameter over time). The use of trended data and interconnection among parameters and variables (multivariate), such as medications and laboratory data, is more clinically meaningful than a given observation in a specific time period. These have not yet been extensively examined [26,27].

Additionally, the Information Center has capabilities of storing different types of ECG and non-ECG waves in graphic and tabular formats, but this valuable information is stored separately from the audit log. The waveform file can be only printed and not stored or exported in e-format. Storing the waveforms information along with each alarm, especially for lethal alarms, would be valuable for classifying false versus actionable alarms.

### Limitations

Our analysis and audit log description represents the offering of one vendor. Although this particular vendor is one of the largest physiologic monitor vendors, the capabilities of other cardiac monitoring devices from other vendors may be different.

### Conclusions

The majority of modern medical devices such as cardiac monitors, smart infusion pumps, and ventilators are capable of automatically logging data. The audit log provides an objective, detailed data source of recorded alarms’ events and types and user’s actions. Unfortunately, this capability is not well utilized in research and quality initiatives. The information presented in this paper may encourage providers, clinicians, and researchers to use audit logs more frequently in research and performance improvement studies.

Despite current challenges in data storage, retrieval, and analysis, large digitalized clinical datasets hold great promise for safety and quality of care. Vendors, clinicians, researchers, and professional organizations should work closely to identify the most useful format and type of clinical data to expand medical devices’ log capacity.

## Abbreviations

ABP: arterial blood pressure
AFIB: atrial fibrillation
Desat: desaturation
ECG: electrocardiographic
HL7: Health Level Seven
HR: heart rate
ICU: intensive care unit
INOP: inoperative
MAUDE: Manufacturer and User Facility Device Experience
MRN: medical record number
NBP: noninvasive blood pressure
NPSG: National Patient Safety Goal
PAP: pulmonary artery pressure
PVC: premature ventricular contraction
RR: respiratory rate
SpO2: peripheral capillary oxygen saturation
STE: ST-segment elevation
